# L1CAM is not a reliable predictor for lymph node metastases in endometrial cancer, but L1CAM positive patients benefit from radiotherapy

**DOI:** 10.7150/jca.59283

**Published:** 2021-09-03

**Authors:** Deborah Zeiter, Tatjana Vlajnic, Andreas Schötzau, Viola Heinzelmann-Schwarz, Céline Montavon

**Affiliations:** 1 Gynaecological Cancer Centre, Hospital for Women, University Hospital Basel and University of Basel, 4031 Basel, Switzerland.; 2 Institute of Pathology, University Hospital Basel and University of Basel, 4031 Basel, Switzerland.; 3 Ovarian Cancer Research, Department of Biomedicine, University Hospital Basel and University of Basel, 4031 Basel, Switzerland.

**Keywords:** L1CAM, lymph node metastasis, endometrial cancer, adjuvant therapy, survival

## Abstract

**Purpose:** Several studies evidenced the potential of L1CAM as a prognostic marker in endometrial cancer. The aim of this study was to investigate whether L1CAM can predict lymph node metastasis and could therefore be used preoperatively to identify patients with low to high-intermediate risk endometrial cancer who would profit from a lymphadenectomy and an adjuvant treatment. To avoid unnecessary morbidity, de-escalating strategies are still required.

**Methods:** Immunohistochemistry for L1CAM was performed on curettage or hysterectomy specimens from 212 patients diagnosed with endometrial cancer who were treated at the University Hospital Basel during 2011-2019. L1CAM expression was correlated with clinicopathological features such as histological subtype, FIGO stage, lymph node metastasis, lymphadenectomy, adjuvant treatment and outcome.

**Results:** Using a cut off ≥10%, L1CAM was positive in 41/212 patients (19.3%) and negative in 171/212 patients (80.7%). L1CAM was associated with high-risk features such as non-endometrioid histology, high tumour grade, and high FIGO stage. There was no significant correlation between L1CAM expression and lymph node metastasis. However, patients with L1CAM positive tumours showed improved disease-specific survival if treated with adjuvant radiotherapy.

**Conclusion:** Although L1CAM expression pointed towards aggressive tumour biology, preoperative L1CAM analysis did not add any substantial predictive information regarding lymph node metastasis in low to high-intermediate risk groups. Therefore, L1CAM status is not suitable to tailor the surgical algorithm for lymph node staging. Nevertheless, our results suggest that L1CAM could be used as a predictive biomarker to select patients who may benefit the most from adjuvant radiotherapy.

## Introduction

Endometrial cancer is the most common gynaecological cancer in the Western World and the second most common worldwide 1. In general, prognosis of early stage endometrial cancer is very good. Nevertheless, a small number of patients experience recurrence and poor survival 2. The current clinical challenge is to identify those patients at high risk for recurrence as they have a significantly worse prognosis and adapt their surgical and adjuvant treatment accordingly 2.

Historically, endometrial carcinoma was divided into two subgroups based on clinical, pathological and molecular characteristics 3. Type I endometrial carcinoma attributes are pre- to peri-menopausal women, obesity, oestrogen-dependence, low grade histological features, and favourable prognosis. Type II endometrial carcinoma are often oestrogen-independent, heterogenous and poorly differentiated tumours, and show worse outcome. However, this stratification is insufficient to identify patients at risk for relapse and metastatic disease 3-6.

The standard therapy for women with endometrial cancer consists of abdominal or laparoscopic hysterectomy and bilateral salpingo-oophorectomy, with or without lymphadenectomy depending on tumour aggressiveness. The presence of lymph node metastasis is one of the most important prognostic factors for poor outcome. The indication for an adjuvant treatment (radiotherapy, brachytherapy, and/or chemotherapy) is based on the presence of clinicopathological risk factors including FIGO-stage, tumour grade, histological type, depth of myometrial invasion and presence of lympho-vascular space invasion (LVSI). Using this risk stratification, patients are divided into five risk categories from “low” to “advanced metastatic” according to the European recommendation of the ESMO-ESGO-ESTRO consensus conference of 2016 7.

While some of the above mentioned risk factors can only be assessed following surgical treatment, robust preoperative markers to identify patients who would benefit the most from lymphadenectomy are still lacking 7-9. While detection of lymph node metastasis supports decision making for an optimal adjuvant treatment, the effect of lymphadenectomy on overall and disease-free survival remains uncertain 10-12. Furthermore, extensive surgery and lymphadenectomy are associated with increased complication and morbidity rates 13. Sentinel lymph node assessment is less invasive, but can be challenging in obese patients 14.

Identification and implementation of genomic features and molecular markers could contribute to a more tailored therapy and even become more important than the information on the nodal status 15. Studies on molecular marker-integrated risk profiling are ongoing, but have not entered into routine clinical practice 16.

L1 cell adhesion molecule (L1CAM), a membrane glycoprotein of the immunoglobulin family, is involved in cancer progression, invasion and metastasis 17-19. L1CAM expression has been associated with poor clinical outcome in a variety of tumours, suggesting a role as prognostic marker 18-20. In endometrial cancer, L1CAM expression is associated with myometrial invasion, cervical involvement, positive LVSI, positive lymph nodes and poor overall survival 21-23. Furthermore, it is associated with worse prognosis in early-stage endometrial cancer, indicating the need for adjuvant treatment in these patients 24. Owing to the fact that marker testing on curettage samples has proven equally reliable as on hysterectomy specimens 25, 26, it might be proposed to use L1CAM in the preoperative risk stratification to predict lymph node metastasis in endometrial cancer. However, L1CAM expression and its usefulness in the preoperative setting is not yet established. Therefore, we investigated the value of L1CAM to predict a more aggressive phenotype with tendency to lymphogenic spread and thus the necessity of tailoring surgical management and receiving adjuvant therapy. We investigated this marker in a cohort of patients with endometrial cancer, including a non-high-risk subgroup according to the ESMO-ESGO-ESTRO classification of 2016. Furthermore, we analysed the association of L1CAM with other established risk factors and the outcome of our patients.

## Materials and Methods

### Patients

**Figure 1:**
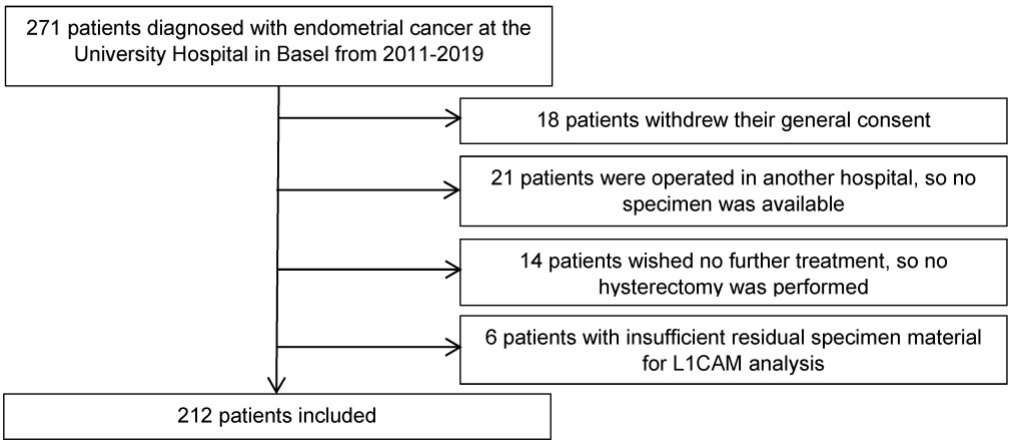
Flow diagram of the excluded patients.

We retrospectively analysed the clinicopathological data from 271 patients diagnosed with endometrial cancer, who were treated at the University Hospital Basel between 2011 and 2019. Eligible patients were at least 18 years old and had endometrial cancer with sufficient tissue for L1CAM assessment in curettage and/or hysterectomy specimen. The final surgical procedure included at least a hysterectomy at our hospital to confirm the histological diagnosis in our pathology department (35 patients excluded). For 6 patients, the residual specimen material was insufficient for immunohistochemical analysis. Eighteen patients were also excluded as they withdrew their general consent. The final cohort included 212 patients (Figure 1).

Baseline demographics, disease characteristics, and follow-up data of all patients were collected and analysed. Additional to L1CAM, the following parameters were included: age, FIGO stage, histology, grade, LVSI, lymph node metastasis, oestrogen receptor (ER) and progesterone receptor (PR) status. Disease recurrence was diagnosed on a histological basis (biopsy) or by radiological confirmation according to the RECIST (version 1.1) 27 criteria. Lymphadenectomy, including sentinel lymphadenectomy, was performed as recommended by international guidelines. Furthermore, we registered all proceeded adjuvant therapies, chemotherapy and/or radiotherapy, including external beam radiotherapy and vaginal vault brachytherapy. The recommendations were based on international data and current guidelines and were adapted according to patient's co-morbidities and wishes. Patient characteristics are shown in Table 1.

Ethics approval was obtained from the Ethical Committee of Nordwest- und Zentralschweiz, Switzerland (EKNZ 2020-00753). The whole study was performed according to the Declaration of Helsinki as well as local laws and regulations.

### L1CAM analysis und immunohistochemistry

For immunohistochemistry, tissue sections of 4 μm were cut from formalin-fixed, paraffin-embedded blocks and mounted on Superfrost slides. Immunohistochemical analysis was performed on the BenchMark Ultra automated immunostaining system (Ventana Medical System Inc., Tucson, AZ). The slides were pretreated with Cell Conditioning 1 (CC1, Ventana Medical System Inc.) for 24 minutes and then incubated for 24 minutes with primary L1CAM mouse monoclonal antibody (purified anti- CD171 (L1) antibody clone 14.10, 1:100 diluted, Biolegend, San Diego, CA, USA). DAB was used as chromogen, and counterstaining was performed with Hematoxylin.

All samples from curettage (n=139) and hysterectomy specimens (n=200) were histologically evaluated by a gynaecologic pathologist (TV) and blinded for clinical outcome data. L1CAM expression analysis (n=212) was performed on curettage specimens (n=139) and if not available on hysterectomy specimens (n=73), listed in the supplementary materials (Table S1). Membranous staining of any intensity in tumour cells was considered positive. The percentage of positive tumour cells was estimated and scored from 0% to 100%. Staining of the nerves within the specimens served as internal positive control.

**Figure 2:**
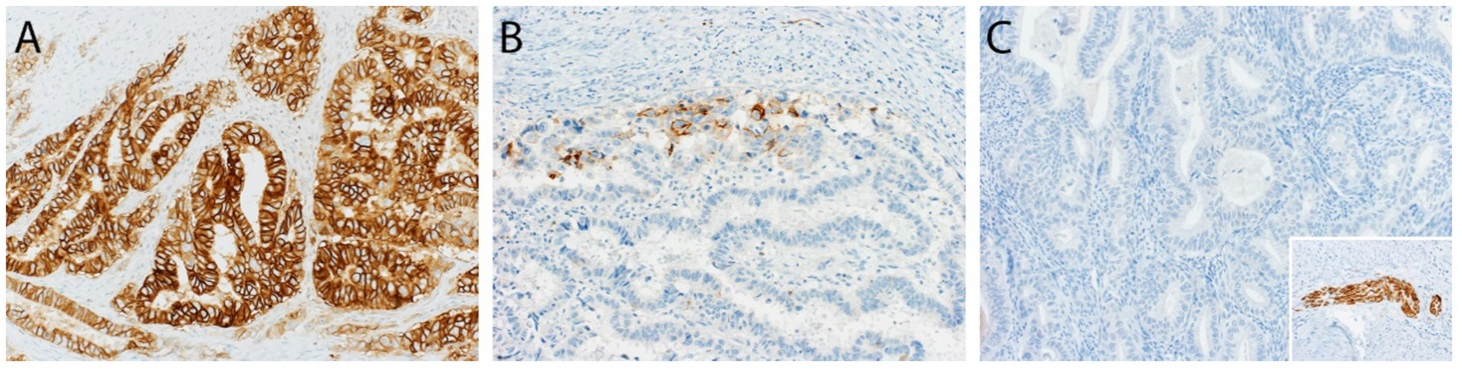
** Representative samples of various L1CAM expression patterns in three different cases of endometrioid endometrial carcinoma. A.** Diffuse L1CAM expression in > 80% of tumour cells. **B.** Focal L1CAM expression in 5% of tumour cells. **C.** Complete absence of L1CAM expression. Inlet: positive internal control in a nerve within the specimen (A-C, original magnification 200x).

A cut-off of ≥10% was used to define a tumour L1CAM-positive or -negative, based on previously published studies 22, 24, which determined the optimal threshold for L1CAM positivity by unpruned classification and regression decision tree and verified it with a 10-fold cross-validation 24. Representative samples of different L1CAM expression patterns in patients with endometrial carcinoma are presented in Figure 2. L1CAM was also reported and analysed as a continuous value (in percentage), as there has been much discussion in the literature about the arbitrary aspects of a 10% threshold.

### Risk stratification

Risk stratification of endometrial cancer was estimated based on histological subtype and tumour grade on curettage specimens, preoperative imaging (e.g. transvaginal ultrasound), as well as intraoperative frozen section analysis (to assess depth of myometrial invasion). This reflected the pre- and intraoperative knowledge of the surgeon. Consecutively, patients were divided into two risk groups: the first group including patients from “low to high-intermediate” risk groups and the second group including patients in the “high to advanced-metastatic” risk groups based on ESMO-ESGO-ESTRO classification 7 (Table 2). As preoperative LVSI was mostly unknown, the exact subdivision in a low, intermediate, or high-intermediate group was often not possible, hence a dichotomous classification was performed.

### Statistical analysis

#### Study population and L1CAM

Descriptive statistics was used to describe the study population as well as to distinguish between L1CAM positive and negative patients. Clinicopathological patients' characteristics of L1CAM positive and negative patients were compared with Mann-Whitney U-test for continuous or ordinal variables and with Pearson's Chi-Square test for categorical variables. Fisher's exact test was applied if there were less than 5 counts in a cell.

#### L1CAM as predictor for lymph node metastasis

Multivariable logistic regression models were created to assess the potential of L1CAM and other parameters to predict lymph node invasion preoperatively. To quantify the predictive quality of the models, the area (AUC) under the receiver operating curve (ROC) was estimated. This also included sensitivity, specificity, negative predictive values as well as positive predictive values for several cut-offs.

#### L1CAM as prognostic marker (L1CAM and further association)

Furthermore, the association between L1CAM (cut-off 10) and other high-risk features, such as advanced FIGO-stage, non-endometrioid histology or positive LVSI were tested using Chi-Square test or Fisher's exact test for categorical variables and Wilcoxon test for metric or ordinal variables.

#### L1CAM and time to event analysis

The recurrence free survival (RFS) was calculated from the date of diagnosis to the last date of progression free follow-up. The disease specific survival (DSS) was calculated from the date of diagnosis to death from disease. Deaths of unknown cause or other than disease were censored. Survival curves were calculated using the Kaplan-Meier method. Additionally, 5-year RFS and DSS were presented with a 95% confidence interval (CI). The log-rank test was used to compare the survival curves. Cox regression was performed to present univariable and multivariable predictions (with covariates). Results are being presented as hazard ratios with corresponding 95% CI's and p-values.

In order to estimate the effect of L1CAM on therapies concerning RFS and DSS, the interaction between L1CAM and adjuvant therapies was additionally included in the Cox-regression. Results are reported as p-value of the interaction.

All statistical tests were two-sided and statistical significance was set at p<0.05. The analyses were performed using the statistical program R version 4.0.0.

## Results

### Study population, risk group stratification and L1CAM

From 2011 to 2019, 212 patients with endometrial cancer diagnosed at the University Hospital in Basel were included in the study (Figure 1). The median age in the study population at diagnosis was 66.0 years [58.0;76.0]. All stages of disease were included in this study as following: stage I 149 (70.3%) patients, stage II 19 (8.96%), stage III 28 (13.2%) and stage IV 16 (7.5%) patients. There were 61 (28.8%) patients with grade 1 tumours, 87 (41%) with grade 2 and 64 (30.2%) with grade 3 tumours. Histological subtypes of endometrial carcinoma comprised 181 (85.4%) endometrioid, 16 (7.55%) serous, 2 (0.94%) clear cell, 10 (4.72%) carcinosarcoma and 3 (1.41%) other classified (1 neuroendocrine carcinoma, 2 dedifferentiated carcinomas). LVSI was present in 57 (26.9%) cases and negative in 155 (73.1%) cases. Lymphadenectomy was performed in 110 (51.9%) patients, 88 (80.0%) of which had negative lymph nodes and 22 (20.0%) had at least one affected lymph node. 102 (48.1%) patients had no examination of lymph nodes (Table 1).

We stratified our patients in risk categories according to the ESMO-ESGO-ESTRO classification of 2016 as following: 71 (33.5%) patients were in the low-risk group, 31 (14.6%) in the intermediate, 22 (10.4%) in the high-intermediate, 65 (30.7%) in the high, and 23 (10.8%) in the advanced metastatic risk group. When using the dichotomous stratification, 124 (58.5%) patients were in the low to high-intermediate risk group and the remaining 88 (41.5%) patients were in the high to advanced-metastatic risk group. Nearly half of the patients (n=114, 53%) required an adjuvant therapy, including vaginal brachytherapy (n=73, 34.43%), pelvic external beam radiotherapy (n=37, 17.45%) and/or chemotherapy (n=59, 27.83%). These treatment modalities might also have been used in a combined fashion. Local recurrences were observed in 9 (4.25%) patients whereas distant recurrences were more frequent and occurred in 20 (9.43%) patients. L1CAM was positive in 41 (19.3%) patients and negative in 171 (80.7%) patients.

### L1CAM as predictor for lymph node metastasis

**Figure 3:**
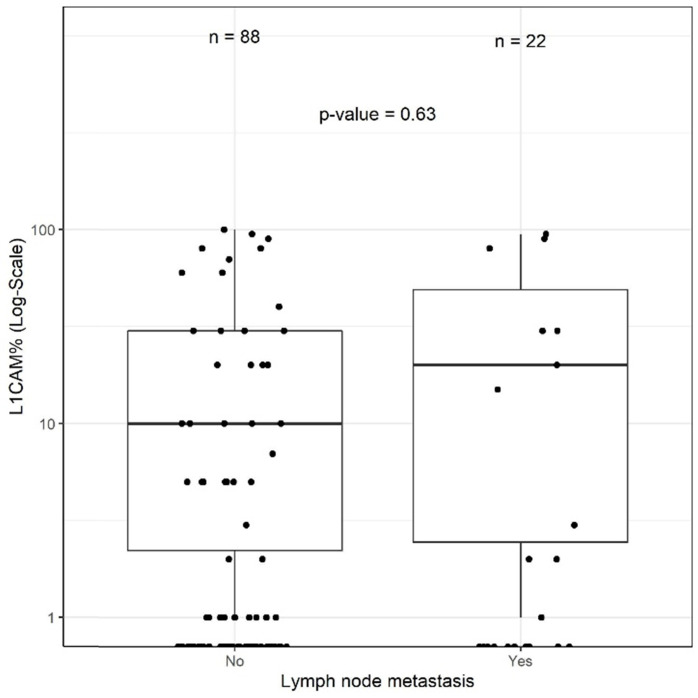
** L1CAM and lymph node.** No significant correlation between L1CAM and lymph node status (p=0.63) could be shown in patients with positive (n=22) and negative (n=88) lymph nodes.

Of the 110 (51.9%) patients receiving lymphadenectomy, lymph node metastases were detected in 22 (20.0%) patients. Of those 22 patients, 15 (68.2%) were L1CAM negative and 7 (31.8%) L1CAM positive. In 88/110 (80.0%) patients, the examined lymph nodes were negative. Out of these patients without lymph node metastasis, 66 (75%) were L1CAM negative and 22 (25%) L1CAM positive. Using the 10% threshold for L1CAM positivity, no significant association between L1CAM expression and lymph node metastasis was found (p=0.705). The predictive value was analysed for the entire cohort as well as within the prognostic subgroups without showing any discriminatory capacity. As different cut-offs had been previously discussed 21, 28, 29, we additionally performed a continuous analysis for L1CAM expression. Logistic regression did not show any significant influence on L1CAM in percent (OR 1.01 [0.99;1.02], p=0.357) (Figure 3). The subsequent ROC curve analysis did not show any predictive ability of L1CAM (AUC 0.530) regarding lymph node metastases either.

### L1CAM and its association with further prognostic factors

L1CAM was associated with factors known to be related to a more aggressive behaviour, such as higher FIGO stage (p=0.009), non-endometrioid histology (p<0.001), high tumour grade (p<0.001), and negative ER and PR status (p<0.001). These results were significant and independent of the L1CAM threshold, reported here are results with the 10% cut-off. Moreover, was L1CAM significantly associated with LVSI (p=0.001) when using L1CAM as a continuous variable, while only a trend was observed (p=0.079) when setting the threshold at 10%. Expectedly, was L1CAM associated with the risk categories according to the ESMO-ESGO-ESTRO classification of 2016.

### Time to event analysis in relation to L1CAM

**Figure 4:**
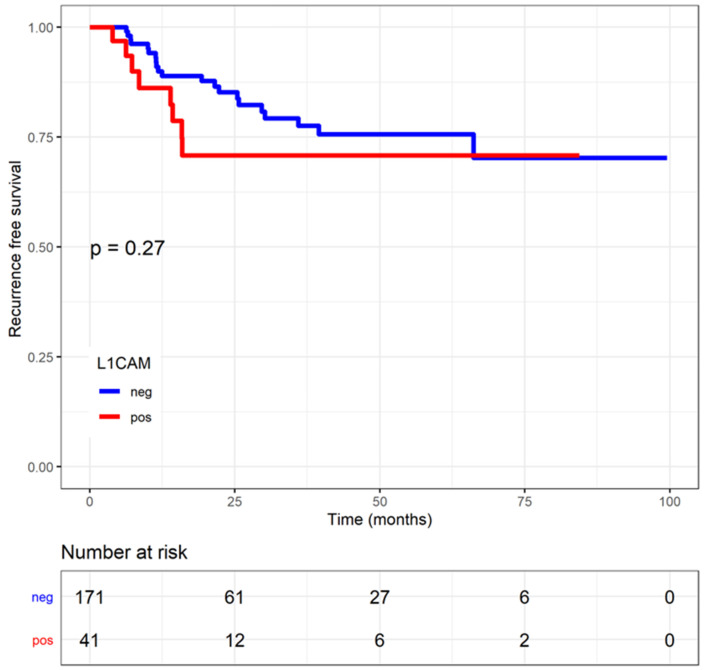
** Regression free survival (RFS) in endometrial cancer patients stratified by L1 cell adhesion molecule (L1CAM) expression Kaplan Meier curve of the RFS.** No difference could be shown between L1CAM positive or negative groups. Log rank test p=0.268.

**Figure 5:**
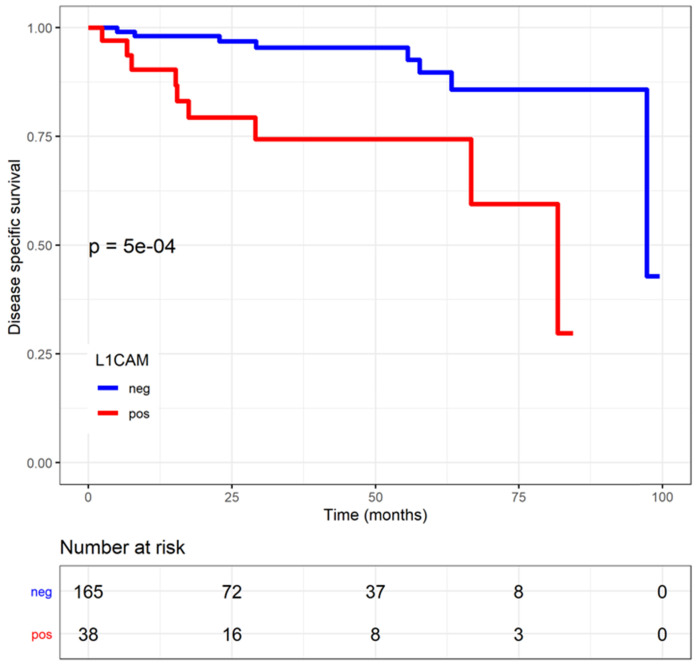
** Disease specific survival (DSS) in endometrial cancer patients stratified by L1 cell adhesion molecule (L1CAM) expression.** L1CAM negative patients showed a significant better DSS. Kaplan Meier curve of the DSS, Log rank test p<0.001.

The median follow-up was 1.37 years (0.20;3.57). Patients with L1CAM positive tumours showed a shorter disease-specific survival (DSS) (L1CAM continuous: HR=1.03, CI: 1.01-1.04, p<0.001; L1CAM 10% threshold: HR=4.89, CI: 1.82-13.18, p<0.001). Moreover, was L1CAM expression associated with shorter recurrence-free survival (RFS) but only when using L1CAM as a continuous variable (L1CAM continuous: HR=1.02, CI: 1-1.03; p=0.006; L1CAM 10% threshold: HR=1.58; CI: 0.70-0.57, p=0.27) (Figures 4 and 5). DSS was significantly shorter in patients with increasing stage, grade of tumour, non-endometrioid histology and LVSI positivity. However, in multivariate analysis only FIGO stage remains an independent prognostic factor for poorer DSS, when the analysis is adjusted for stage, grade, histology, LVSI and L1CAM (Table 3).

We analysed the patient outcome according to adjuvant therapies applied, including external beam radiotherapy (EBRT), vaginal brachytherapy (VBT), or chemotherapy, and their correlation to L1CAM. The higher L1CAM was expressed, the more patients showed a survival benefit (RFS, DSS) from any adjuvant therapy, being external beam radiotherapy (EBRT), vaginal brachytherapy (VBT), or chemotherapy. Patients with L1CAM positive tumours who received adjuvant therapy showed longer DSS than the ones without adjuvant therapy (p=0.0055, L1CAM continuous; p=0.0426, L1CAM 10%). After adjuvant therapy a 79% improvement of DSS (HR 0.21, 95% CI: 0.04-1.10) was observed. The 5-year DSS for patients with L1CAM positive tumours was 78% after therapy versus 38% without adjuvant treatment. Only a trend was observed for RFS (p=0.066, L1CAM continuous). In L1CAM negative patients, adjuvant therapies had no impact on DSS.

**Figure 6:**
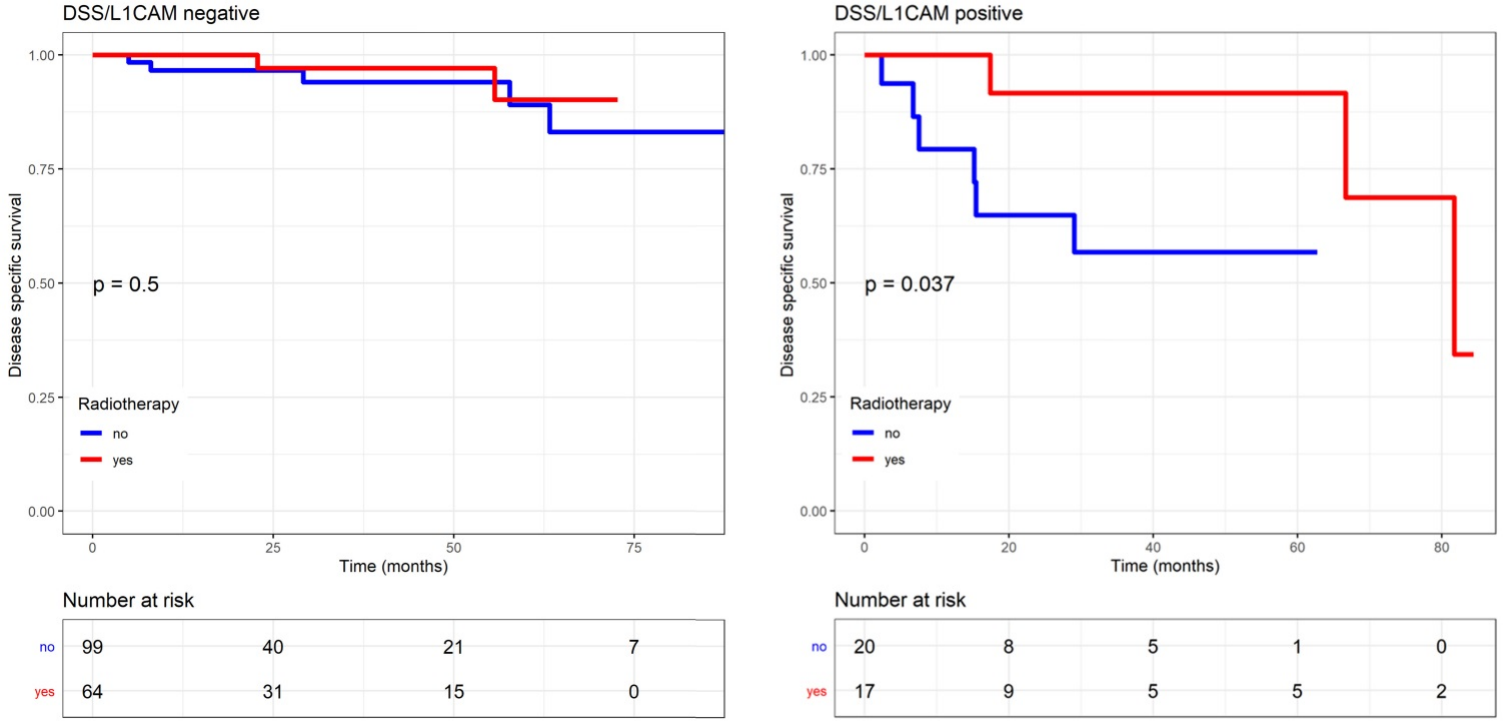
** Endometrial-cancer related survival by L1CAM with or without radiotherapy (including EBRT and VBT).** L1CAM positive patients benefit from radiotherapy, as they showed a significant better DSS after treatment compare to no radiotherapy (Log rank test p=0.037). Kaplan Meier curve of the DSS. EBRT (external beam radiotherapy); DSS (disease specific survival); L1CAM (L1 cell adhesion molecule); VBT (vaginal brachytherapy).

The effects of the adjuvant therapy were more closely analysed, and no interaction with chemotherapy was observed. However, DSS significantly improved if L1CAM positive patients received radiotherapy (including EBRT and VBT) compared to those who did not, as reported in the Kaplan Meier analysis (p=0.037, log rank) (Figure 6). For L1CAM positive patients, we observed an 86% improvement of DSS (HR 0.14, 95% CI: 0.02-1.19) after radiotherapy. L1CAM positive patients without radiotherapy are more likely to die earlier, as their 5-year DSS was 57% compared to 92%. DSS benefit of radiotherapy was also observed in the endometrioid L1CAM positive subgroup (p=0.036, L1CAM continuous). A further subgroup analysis was not carried out due to a small number of events and the questionable data validity.

## Discussion

In order to personalize the treatment of endometrial cancer patients, reliable markers are needed to help to tailor adjuvant treatment as well as the surgical management. Definite indications for lymphadenectomy are missing as this procedure remains controversial 10-12, with a questionable benefit in some risk populations and a considerable morbidity. As L1CAM has been associated with a more aggressive tumour biology and worse outcome, we investigated whether L1CAM can improve preoperative decision-making strategies and allows for avoidance of lymphadenectomy in a selected population of patients with endometrial cancer. We also investigated the benefit of tailored therapy following L1CAM assessment for stratification in our interdisciplinary tumour board.

L1CAM is a widely available and easily evaluable immunohistochemical marker, which could complement existing preoperative risk stratification in clinical practice. A high negative predictive value of L1CAM for lymph node metastasis in a non-high-risk-patients cohort would allow for consideration of de-escalating surgical procedures. This strategy would be particularly important for elderly and/or polymorbid patients with notable peri- and postoperative risks for complications. Especially in cases where a sentinel lymph node assessment fails, a systematic lymphadenectomy is performed, resulting in a considerable morbidity for some of these patients. The balance between benefit and risk of a lymphadenectomy remains a surgical burden that can be relieved but not omitted by following the guidelines. We retrospectively investigated the impact of the implementation of L1CAM into our risk assessment at the tumour board regarding different risk groups. We stratified the patients based on preoperative information, thus mimicking the clinical situation of the surgeon in two groups: The first group including patients from low to high-intermediate risk and the second group patients from high to advanced-metastatic risk. When considering the entire cohort or the risk groups, L1CAM was not a predictor for lymph node metastasis. There was no significant association when using different cut-offs nor a continuous analysis for L1CAM. Consequently, L1CAM did not improve current risk stratification when adding additional parameters such as tumour grade, age, histology, LVSI, or ER and PR status. The use of L1CAM in preoperative risk stratification is differently discussed in the literature. Our results are in line with previous findings of Pasanen et al. 23 who failed to show an improvement of preoperative risk stratification using L1CAM. On contrary, Tangen et al. 29 stated that L1CAM was an independent predictor of lymph node metastasis. These differences could be explained by the different scoring methods and cut-offs being used for L1CAM. Particularly, it seems inadequate to only consider intensity for staining. Our data indicate that preoperative L1CAM assessment using percentage assessment is not helpful in decision making regarding lymphadenectomy. Nevertheless, our data confirm previously demonstrated associations of L1CAM expression and other high-risk features such as FIGO stage 30, tumour grade 30-32, histological subtypes 30, 31, LVSI 21, 30, 33, and ER and PR status 34. We showed that L1CAM is a robust marker that indicates patients at higher risk for distant relapse. These findings are in accordance with previous reports 24, 35.

Thus, the urgent need for helpful markers makes clinicians more prone to guideline deviations, while integrating well known prognostic markers into clinical decisions without evidence of clinical benefit. In the era of precision medicine, this represents a real source of potential pitfalls. Biomarkers - such as L1CAM - should be tested within a selection algorithm in prospective trials before clinical implementation.

L1CAM was shown to be a predictor for poorer outcome 24, 28, 35, 36. In our cohort, L1CAM expression was associated with an increased likelihood of death from endometrial cancer. Moreover, in the analysis of L1CAM as continuous variable, RFS was also negatively affected. Our findings suggest that the influence of L1CAM is even stronger on DSS than on RFS, which also seems to be reflected in previous reports 37, 38. Although L1CAM was not an independent prognostic factor for poorer DSS, after adjustment for stage, grade, histology and LVSI in our cohort, it should be noticed that only the FIGO stage showed an independent association. None of the other factors showed an effect strong enough to predict survival outcome significantly and independently. Stage of disease remains a strong prognostic factor, that could indeed weaken the effect of the other predictors.

More importantly, DSS significantly improved in L1CAM positive patients who received any type of adjuvant therapy (chemotherapy or radiotherapy). This benefit is reflected in a better 5-year DSS of 78% versus 38%. Furthermore, our results indicate that DSS of L1CAM positive patients was mainly influenced by radiotherapy (EBRT or VBT). Those patients were more likely to live longer, with a 5-year DSS of 92% compared to 57%. The benefit of radiotherapy was also observed in patients with endometrioid L1CAM positive tumours. Due to small numbers, we did not analyse the effect of EBRT and VBT separately. But in this context, it is worthwhile to mention the results of the PORTEC-2 trial, which confirmed VBT as standard treatment for high-intermediate risk patients. However, Wortman et al. showed that EBRT seems to provide a better control than VBT in high-intermediate risk patients with unfavourable risk factors, such as L1CAM expression 39. In our study, L1CAM negative patients did not benefit from radiotherapy.

As L1CAM is associated with high-risk features, a more aggressive biological behaviour is expected and consecutively a higher likelihood to die of disease. However, the Kaplan Meier curves demonstrate a surprising benefit of the adjuvant therapy in general, and radiotherapy in particular. The indication for any adjuvant therapy was based on a risk stratification (including the risk factors mentioned above) and the recommendation of our interdisciplinary tumour board according to international guidelines. The indication was given without considering the L1CAM status.

These findings suggest that L1CAM should be used in the postoperative setting to detect patients who could benefit from radiotherapy. The integration of L1CAM in the decision-making strategy for adjuvant therapy needs to be investigated in further studies. Because of its retrospective analysis, this study is limited by potential biases, due to patient selection or incomplete data acquisition. Furthermore, our study cohort is heterogeneous and has relatively few patients, which limited our power to study the prognostic significance of L1CAM in specific subgroups of tumour. Due to the small number of events and cases, outcome analyses remain exploratory.

## Conclusion

In conclusion, L1CAM alone or its integration with established clinicopathological features in endometrial cancer does not improve risk stratification with potential therapeutic implications in the lymph node surgery. L1CAM is not a reliable predictor for lymph node metastases, and therefore preoperative L1CAM assessment is not recommended. Finally, our results suggest that L1CAM could be used in an adjuvant setting as a predictive biomarker to select patients who would benefit the most from an adjuvant therapy, particularly from any form of radiotherapy. A prospective validation needs to be performed within a trial setup.

## Supplementary Material

Supplementary table.

## Figures and Tables

**Table 1:** Clinicopathologic patient characteristics according to L1CAM expression (n=212)

	Total (N=212)	L1CAM negative (<10%) (N=171)	L1CAM positive (≥10%) (N=41)	p-value	number
Age at diagnosis (y)	66.0 [58.0;76.0]	66.0 [58.0;76.0]	66.0 [60.0;73.0]	0.790	212
**Tumour grade**				<0.001	212
1	61 (28.8%)	59 (96.7%)	2 (3.3%)		
2	87 (41.0%)	79 (90.8%)	8 (9.2%)		
3	64 (30.2%)	33 (51.6%)	31 (48.4%)		
**Histological subtype**				<0.001	212
Endometrioid	181 (85.4%)	162 (89.5%)	19 (10.5%)		
Serous	16 (7.55%)	2 (12.5%)	14 (87.5%)		
MMMT	10 (4.72%)	5 (50%)	5 (50%)		
Clear cell	2 (0.94%)	0 (0.00%)	2 (100%)		
Others	3 (1.42%)	2 (66.7%)	1 (33.3%)		
**FIGO stage**				0.022	212
I	149 (70.3%)	127 (85.2%)	22 (14.8%)		
II	19 (8.96%)	15 (78.9%)	4 (21.1%)		
III	28 (13.2%)	21 (75%)	7 (25%)		
IV	16 (7.5%)	8 (50%)	8 (50%)		
**LVSI**				0.079	212
Negative	155 (73.1%)	130 (83.9%)	25 (16.1%)		
Positive	57 (26.9%)	41 (73.7%)	16 (26.3%)		
**ESMO risk classification 2016 7**				<0.001	212
Low	71 (33.5%)	68 (95.8%)	3 (4.2%)		
Intermediate	31 (14.6%)	29 (93.5%)	2 (6.5%)		
High- intermediate	22 (10.4%)	18 (81.8%)	4 (18.2%)		
High	65 (30.7%)	41 (63.1%)	24 (36.9%)		
Advanced metastatic	23 (10.8%)	15 (65.2%)	8 (34.8%)		
**Adapted risk groups (for surgery)**				<0.001	212
Low to high-intermediate	124 (58.5%)	115 (92.7%)	9 (7.3%)		
High to advanced metastatic	88 (41.5%)	56 (63.6%)	32 (36.4%)		
**Lymph node metastasis**				0.705	212
Negative	88 (41.5%)	66 (75%)	22 (25%)		
Positive	22 (9.4%)	15 (68.2%)	7 (31.8)		
Lymph node status not available (pNx)	102 (48.1%)	90 (88.2%)	12 (11.8%)		
**ER (%)**	80.0 [40.0;95.0]	90.0 [70.0;95.0]	5.00 [0.00;50.0]	<0.001	145
**PR (%)**	80.0 [21.2;95.0]	80.0 [50.0;95.0]	5.00 [0.00;55.0]	<0.001	126
**Recurrence**				0.033	212
Local	9 (4.2%)	9 (100%)	0 (0.00%)		
Distant	20 (9.4%)	12 (60%)	8 (40%)		
No recurrence	183 (86.3%)	150 (82%)	33 (18%)		
**Adjuvant therapy**				0.002	212
None	95 (44.8%)	86 (90.5%)	9 (9.5%)		
Any	114 (53.8%)	83 (72.8%)	31 (27.2%)		
Not available	3 (1.42%)	2 (66.7%)	1 (33.3%)		

**Table 2:** Risk groups by the ESMO-ESGO-ESTRO^1^ to guide adjuvant therapy

Risk group	Description	Newly formed risk groups based on ESMO-ESGO-ESTRO
Low	Stage IA endometrioid + grade 1-2 + LVSI negative	Risk group 1
Intermediate	Stage IB endometrioid + grade 1-2 + LVSI negative	
High-intermediate	Stage IA endometrioid + grade 3, regardless of LVSI status Stage I endometrioid + grade 1-2, LVSI unequivocally positive, regardless of depth of invasion	
High	Stage IB endometrioid + grade 3, regardless of LVSI status Stage II & stage III endometrioid with no residual disease Non endometrioid (serous, clear cell, undifferentiated carcinoma, carcinosarcoma or mixed <10%)	Risk group 2
Advanced metastatic	Stage III with residual disease Stage IVA &IVB	

**Table 3:** Univariate and multivariate survival analysis (DSS) of L1CAM and established clinicopathological risk factors

	all	HR (univariate)	HR (multivariate)
**FIGO**			
I	149 (100.0)	-	-
II	19 (100.0)	2.06 (0.18-23.62, p=0.562)	1.42 (0.09-21.33, p=0.800)
III	28 (100.0)	17.65 (2.97-104.98, p=0.002)	25.49 (2.93-221.56, p=0.003)
IV	16 (100.0)	84.75 (15.52-462.72, p<0.001)	135.19 (11.21-1630.81, p<0.001)
**Grade**			
1	61 (100.0)	-	-
2	87 (100.0)	2.29 (0.46-11.38, p=0.311)	0.54 (0.07-4.30, p=0.562)
3	64 (100.0)	6.60 (1.36-32.01, p=0.019)	0.81 (0.08-8.80, p=0.866)
**Endometrioid**			
no	31 (100.0)	-	-
yes	181 (100.0)	0.24 (0.09-0.69, p=0.007)	0.39 (0.08-1.96, p=0.252)
**LVSI**			
0	155 (100.0)	-	-
1	57 (100.0)	6.50 (2.35-17.99, p<0.001)	0.56 (0.12-2.54, p=0.451)
**L1CAM**			
negative	171 (100.0)	-	-
positive	41 (100.0)	4.89 (1.82-13.18, p=0.002)	1.10 (0.28-4.33, p=0.891)

DSS (disease specific survival) ; L1CAM (L1 cell adhesion molecule) ; LVSI (lymph-vascular space invasion); FIGO (Fédération Internationale de Gynécologie et d'Obstétrique).

## Abbreviations

AUC: Area under the curve
CI: Confidence Interval
DSS: Disease specific survival
EBRT: External beam radiotherapy
ER: Oestrogen receptor
ESMO-ESGO-ESTRO: European Society for Medical Oncology - European Society for Gynaecological Oncology - European Society for Radiotherapy and Oncology
FIGO: Fédération Internationale de Gynécologie et d'Obstétrique
HR: Hazard ratio
L1CAM: L1 cell adhesion molecule
LVSI: lymph-vascular space invasion
PR: Progesterone receptor
RECIST: Response Evaluation Criteria In Solid Tumors
RFS: Regression free survival
ROC: Receiver operating characteristic
VBT: Vaginal brachytherapy
