# Supplementary material for: L1CAM is not a reliable predictor for lymph node metastases in endometrial cancer, but L1CAM positive patients benefit from radiotherapy
Source: J Cancer. 2021 Sep 3;12(21):6401–10. doi: 10.7150/jca.59283 (PMC8489141; doi:10.7150/jca.59283)
Supplement: Supplementary file 1 — Supplementary table. [file jcav12p6401s1.pdf]

Number of patients	L1CAM (%)
1	0
2	30
3	1
4	0
5	0
6	0
7	10
8	100
9	3
10	5
11	5
12	50
13	1
14	1
15	0
16	1
17	0
18	0
19	0
20	0
21	10
22	0
23	1
24	0
25	0
26	0
27	0
28	0
29	5
30	30
31	20
32	0
33	0
34	0
35	0
36	0
37	3
38	90
39	0
40	0
41	5
42	80
43	0
44	0
45	0

46	0
47	0
48	0
49	3
50	2
51	90
52	30
53	10
54	0
55	5
56	0
57	1
58	0
59	0
60	0
61	0
62	2
63	1
64	0
65	0
66	0
67	0
68	70
69	0
70	0
71	60
72	95
73	0
74	1
75	0
76	0
77	0
78	0
79	1
80	5
81	0
82	5
83	0
84	0
85	0
86	5
87	5
88	0
89	1
90	0
91	2
92	0

93	0
94	95
95	5
96	0
97	0
98	5
99	0
100	0
101	0
102	0
103	0
104	0
105	0
106	0
107	0
108	0
109	40
110	0
111	30
112	0
113	0
114	1
115	30
116	20
117	2
118	1
119	5
120	0
121	0
122	10
123	1
124	80
125	2
126	2
127	0
128	17.5
129	0
130	0
131	1
132	0
133	5
134	1
135	60
136	30
137	10
138	0
139	20

140	80
141	1
142	0
143	0
144	0
145	0
146	0
147	20
148	10
149	0
150	30
151	0
152	0
153	15
154	0
155	0
156	0
157	0
158	1
159	0
160	0
161	0
162	0
163	50
164	2
165	1
166	80
167	7
168	10
169	1
170	30
171	0
172	15
173	0
174	0
175	0
176	0
177	20
178	0
179	2
180	0
181	0
182	0
183	0
184	0
185	0
186	0

187	90
188	1
189	0
190	0
191	1
192	0
193	1
194	0
195	0
196	0
197	15
198	0
199	0
200	0
201	0
202	0
203	0
204	0
205	40
206	0
207	1
208	0
209	1
210	0
211	0
212	0

Table S1: Used L1CAM values in percent of all included patients
